# On Cayley graphs of 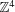


**DOI:** 10.1107/S2053273320007159

**Published:** 2020-07-16

**Authors:** Igor A. Baburin

**Affiliations:** a Theoretische Chemie, Technische Universität Dresden, Bergstraße 66c, Dresden, 01062, Germany

**Keywords:** Cayley graphs, free abelian groups, computational group theory, vertex-transitive graphs, isotopy

## Abstract

The generating sets of  have been enumerated which consist of integral four-dimensional vectors with components −1, 0, 1 and allow Cayley graphs without edge intersections in a straight-edge embedding in a four-dimensional Euclidean space. Owing to computational restrictions the valency of enumerated graphs has been fixed to 10. Up to isomorphism 58 graphs have been found and characterized by coordination sequences, shortest cycles and automorphism groups. To compute automorphism groups, a novel strategy is introduced that is based on determining vertex stabilizers from the automorphism group of a sufficiently large finite ball cut out from an infinite graph. Six exceptional, rather ‘dense’ graphs have been identified which are locally isomorphic to a five-dimensional cubic lattice within a ball of radius 10. They could be built by either interconnecting interpenetrated three- or four-dimensional cubic lattices and therefore necessarily contain Hopf links between quadrangular cycles. As a consequence, a local combinatorial isomorphism does not extend to a local isotopy.

## Abstract 2

Cayley graphs of 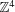 with valency 10 have been enumerated which correspond to generating sets of integral vectors with components −1, 0, 1 and which are embedded in a four-dimensional Euclidean space without edge intersections.

## 1. Introduction   

Cayley graphs provide a helpful tool to ‘visualize a group’ and to derive its properties (*e.g*. defining relations) in an essentially geometric way (*cf*. Löh, 2017 ▸). As usual, we consider a Cayley graph of a group *G* with an inverse-closed finite generating set *S* (such that *S* = *S* ^−1^ 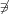 1) as an undirected graph whose vertices correspond to group elements and vertices 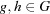 are connected by an edge whenever 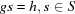. For additive groups (*e.g*. for 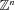, *n* being a positive integer) we may write 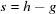.

Cayley graphs of crystallographic groups in a Euclidean plane were treated in detail in a well known book by Coxeter & Moser (1980 ▸). The situation in higher dimensions (> 2) is far from having been completely explored. Although in dimension 3 different enumeration methods indeed produced many Cayley graphs of crystallographic groups relevant to structural chemistry (*e.g*. Fischer, 1974 ▸, 1993 ▸), their potential has never been used in full [some applications are described by Eon (2012 ▸)]. Despite some results on *lattice nets* or *bouquet nets* (Delgado-Friedrichs & O’Keeffe, 2009 ▸; Moreira de Oliveira & Eon, 2014 ▸), the terms adopted by crystallographers for Cayley graphs of 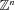, complete enumerations for 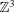 (under fairly natural assumptions) have become available only recently (Power *et al.*, 2020 ▸). Many important properties of Cayley graphs of 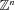 were derived by Kostousov (2007 ▸) which we quote below (Section 2.1).

There exists only one (up to isomorphism) Cayley graph of 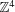 with valency 8 that corresponds to a four-dimensional hypercubic lattice. In this paper we provide a complete catalogue of Cayley graphs of 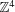 with valency 10 which arise for generating sets of integral vectors with components −1, 0, 1 (loosely speaking, the shortest) and which are embedded in a four-dimensional Euclidean space, *i.e*. free of edge crossings in a straight-edge embedding (in other words, edges intersect at most at common vertices). The restriction to valency 10 in the present study is due to a significant increase in the computational demand for isomorphism checking already for the next possible valency 12, in which case effective invariants are to be developed to quickly distinguish non-isomorphic graphs. The structure of graphs is characterized in terms of coordination sequences and shortest cycles. Additionally, we apply a novel strategy to compute automorphism groups.

## 2. Theoretical background and computational methodology   

### Some properties of Cayley graphs of 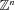   

2.1.

We start by summarizing important facts about Cayley graphs of 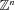. In the following, we associate 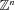 with an additive group of *n*-dimensional integral vectors of an affine Euclidean space 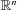.

**Theorem 1**

[Kostousov (2007 ▸), Theorem 3, part (*a*), and Proposition 3.] Let *S* and *M* be generating sets of 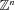 which consist of *n*-dimensional integral *row vectors*. The respective Cayley graphs are isomorphic iff there is a matrix 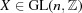 with |det(*X*)| = 1 such that 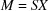.

Theorem 1[Statement theorem1] provides a handy criterion for isomorphism testing by solving a system of linear equations. We note that isomorphism testing by computing canonical forms according to Delgado-Friedrichs (2004 ▸) turns out to be rather expensive in dimensions *n* > 3.

**Theorem 2**

[Kostousov (2007 ▸), Theorem 3, part (*b*); Moreira de Oliveira & Eon (2014 ▸), Theorem 4.1.] The automorphism group of a Cayley graph Γ of 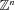, Aut(Γ), is isomorphic to a crystallographic group.

As an immediate consequence we obtain that vertex stabilizers in Aut(Γ) are finite.

Any Cayley graph of 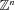 allows a natural embedding in 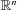, with vertices as nodes of an integral lattice and edges as straight-line segments corresponding to generators. Any automorphism of a graph in this embedding is induced by an affine map of 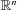. The following theorem provides a group-theoretic condition for when this embedding is free of edge intersections.

**Theorem 3**

[*cf*. Power *et al.* (2020 ▸), Proposition 4.5.] Let Γ be a Cayley graph of 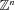 with respect to a generating set *S*, and let Γ be embedded in 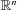 as described above with edges as straight-line segments. Then Γ is free of edge intersections (except at the vertices of Γ) iff 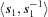 is a maximal rank 1 subgroup of 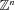 for any 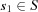 and 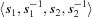 is a maximal rank 2 subgroup of 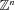 for any 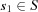 and 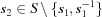.

**Proof**

A pair of intersecting edges of Γ spans a one- or two-dimensional affine subspace. Subgraphs of Γ which correspond to 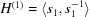 and 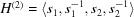 are chains and (topological) square grids, respectively. Let 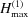 and 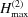 be maximal rank 1 and rank 2 subgroups of 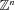 such that 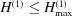 and 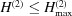. If 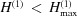, then cosets of 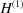 in 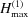 generate collinear chains in Γ running along the direction defined by 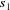. To have only one chain rather than a set of collinear chains implies 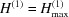. Similarly, if 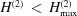, cosets of 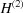 in 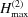 give rise to square grids which are shifted against each other in a two-dimensional plane defined by 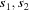 that forces edges to cross. As a consequence, edge intersections do not take place iff subgroups *H* ^(1)^ and *H* ^(2)^ are maximal subgroups of 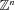 with rank 1 and 2, respectively.

□

**Remark 1**

If the conditions of Theorem 3[Statement theorem3] are fulfilled but subsets of *S* generate non-maximal subgroups of 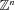 with rank *d* ≥ 3, then an affine subspace of dimension *d* accommodates a finite number of connected components (each of dimensionality *d*) which do not cross each other. This implies the existence of Hopf links between the cycles of a graph (*cf*. Section 3).

**Remark 2**

From Theorem 3[Statement theorem3] it is possible to determine the maximal valency for Cayley graphs of 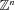 which can be embedded in 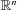 without edge intersections provided the components of generating vectors are restricted to a certain range. For example, if vectors with components −1, 0, 1 are considered, the maximal valency is 6, 14, 30, 62, 126 for *n* = 2, 3, 4, 5, 6, respectively.

### Computation of automorphism groups for Cayley graphs of 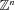   

2.2.

Since any Cayley graph Γ of 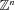 obviously does not show up vertex collisions in a barycentric placement, the method of Delgado-Friedrichs (2004 ▸) [*cf*. also Delgado-Friedrichs & O’Keeffe (2003 ▸) for a less formal exposition] can be used to compute Aut(Γ). Here we have adopted a different strategy that involves a computation of a vertex stabilizer in Aut(Γ) from the local structure of a graph Γ. This strategy is quite general and appears to be very effective for vertex-transitive periodic graphs with finite vertex stabilizers in Aut(Γ).

To facilitate the following discussion, let us establish some notation for graphs and group actions on various sets associated with them.

Let Γ be a connected simple graph with finite valencies of vertices. The distance between vertices *x* and *y*, *d*(*x*, *y*), is defined as the number of edges in a shortest path from *x* to *y*. Then *B*_Γ_(*v*, *r*) = {*x* | *d*(*v*, *x*) ≤ *r*} is the ball with a radius of *r* edges centred at *v*, and 〈*B*_Γ_(*v*, *r*)〉 is the subgraph of Γ induced by *B*_Γ_(*v*, *r*). If there is no ambiguity we shall write *B*(*v*, *r*). A coordination sequence for a vertex *v* is a sequence of integers {|*S*(*v*, *i*)|} where *S*(*v*, *i*) = {*x* | *d*(*v*, *x*) = *i*} is the sphere of vertices at distance precisely *i* from *v*. The automorphism group of Γ, Aut(Γ), is regarded as a group of all adjacency-preserving permutations on the vertex set of Γ. Two (generally non-isomorphic) vertex-transitive graphs Γ_1_ and Γ_2_ are said to be *locally isomorphic* within a ball of radius *r* if 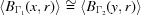. If *G* is a permutation group on a set *X*, then Stab*_G_*(*x*) = {*g* | *xg* = *x*, *g* 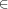 *G*}, *i.e.* the stabilizer of an element 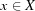 in *G*. If 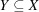 is *G*-invariant, *G^Y^* is the restriction of *G* to a subset *Y*.

**Proposition 1**

[Trofimov (2012 ▸), Section 3.] Let Γ be a connected vertex-transitive graph and *v* be a vertex of Γ. For any non-negative integer *r* there exists a minimal integer ρ(*r*) ≥ *r* such that
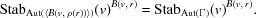
In other words, any automorphism of 〈*B*(*v*, *r*)〉 fixing *v* which can be extended to an automorphism of 〈*B*(*v*, ρ(*r*))〉 can also be extended to an automorphism of Γ.

**Proposition 2**

Let Γ be a Cayley graph of 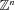. For any vertex *v* of Γ, the stabilizer Stab_Aut__(Γ)_(*v*) acts faithfully on *B*(*v*, 1).

**Proof**

By Theorem 2[Statement theorem2] Aut(Γ) is isomorphic to a crystallographic group. Vertices adjacent to *v* form an *n*-dimensional convex hull that cannot be stabilized pointwise by any crystallographic isometry (or an affine map) of 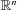.

□

**Proposition 3**

Let Γ be a Cayley graph of a group *G* with respect to a generating set *S*, and *v* be a vertex of Γ. Then Aut(Γ) = 〈*S*, Stab_Aut__(Γ)_(*v*)〉.

For a Cayley graph Γ of 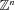 Propositions 1[Statement proposition1] and 2[Statement proposition2] allow us to determine a faithful action of Stab_Aut__(Γ)_(*v*) on *B*(*v*, 1) as a permutation group from the restriction of Stab_Aut(〈*B*(*v*, ρ(1))〉)_(*v*) to *B*(*v*, 1). A practical computation of ρ(1), Stab_Aut__(Γ)_(*v*) and eventually Aut(Γ) (the latter requires Proposition 3[Statement proposition3]) is facilitated by employing the fact that any automorphism of Γ is induced by an affine map of 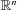 if vertices of Γ are associated with nodes of an integral lattice as done in Section 2.1.

Given an input set *S* of *n*-dimensional integral vectors, the automorphism group of the respective Cayley graph Γ of 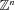, Aut(Γ), can be computed as a matrix group in the following steps (hereafter *v* is the vertex (0, …, 0) of Γ):

(i) For some *k* ≤ ρ(1) generate a finite subgraph 〈*B*(*v*, *k*)〉 of Γ and compute Aut(〈*B*(*v*, *k*)〉).^1^

(ii) Compute generators of Stab_Aut(〈*B*(*v*, *k*)〉)_(*v*)^*B*(*v*, 1)^ as permutations on vertices of *B*(*v*, 1).

(iii) Check (by solving systems of linear equations) if permutations computed at step (ii) are induced by integral *n* × *n* matrices. If so, then *k* = ρ(1) is found. The set *T* of the so-obtained matrices generates an integral matrix representation of Stab_Aut(Γ)_(*v*), and we proceed to step (iv). Otherwise we set *k*: = *k* + 1 and go back to step (i).

(iv) Aut(Γ) is output as a matrix group generated by *S* and *T*: Aut(Γ) = 〈*S*, *T*〉 (elements of *S* and *T* are expressed here as (*n*+1) × (*n*+1) *augmented* matrices).

**Remark**

Recently another method [see Section 3.1 in Bremner *et al.* (2014 ▸)] has come to our attention that allows one to compute Stab_Aut(Γ)_(*v*) [*v* = (0, …, 0)] as an integral matrix group by making use of a positive definite symmetric matrix 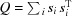, where the sum runs over *column vectors* 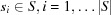. The automorphism group of *Q*, Aut(*Q*), is defined as
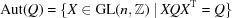
and corresponds to the isometry group of an *n*-dimensional integral lattice with a Gram matrix *Q*. Aut(*Q*) can be computed using the algorithm of Plesken & Souvignier (1997 ▸) as implemented in the *AUTO* program.^2^ Stab_Aut__(Γ)_(*v*) is readily obtained as a *setwise* stabilizer of *S* in Aut(*Q*).

## 3. Results and discussion   

With the above theory in mind, we have implemented in the GAP programming language (GAP, 2019 ▸) the search for generating sets of 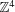 which give rise to Cayley graphs of valency 10 by enumerating quintuples of four-dimensional vectors with components −1, 0, 1. Filtering out generating sets which satisfy our Theorem 3[Statement theorem3] (Section 2.1) and yield isomorphic graphs was done on the fly, and the computation of automorphism groups was implemented in a separate program making use of *nauty* (McKay, 2009 ▸) and the *Cryst* package (Eick *et al.*, 2019 ▸). For checking purposes, automorphism groups were also computed with an alternative method based on the Remark in Section 2.2. The results are gathered in Table 1 ▸. Furthermore, the supporting information contains explicit lists of generators, point symbols (Blatov *et al.*, 2010 ▸) and coordination sequences up to the 15th sphere.

To our knowledge, only three out of the 58 graphs have been known before, namely, #1, #2 and #20 which correspond to primitive hexagonal tetragonal, *I*-centred cubic orthogonal and primitive icosahedral lattices^3^ (O’Keeffe, 1995 ▸). The ‘topological’ diversity of the graphs is very much restricted since they all turn out to be closely related to a five-dimensional (primitive) cubic lattice as shown by point symbols and coordination sequences. This is not accidental since Cayley graphs of 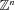 with valency 2(*n*+1) are indeed quotients of an (*n*+1)-dimensional cubic lattice with respect to some rank 1 subgroup (*cf*. Eon, 2011 ▸). Low-dimensional quotients necessarily inherit certain properties from their parent higher-dimensional counterparts.

**Figure 1:**
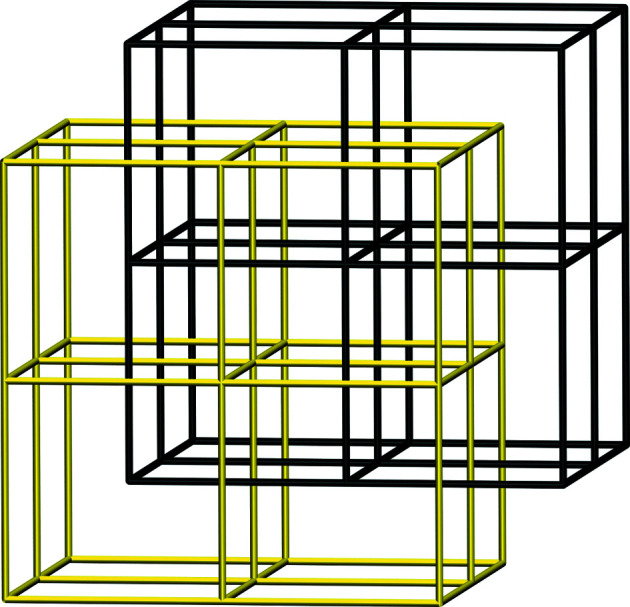
Two interpenetrating primitive cubic lattices in a three-dimensional Euclidean space.

Generating sets for 55 graphs (all except #1, #2 and #20) contain subsets corresponding to non-maximal 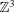 or 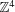 subgroups (or both). This means that quadrangular cycles of the graphs are linked (*cf*. Remark 1[Statement enun1] to Theorem 3[Statement theorem3]). Let us discuss this phenomenon in more detail for six exceptional graphs (#49, #52, #54, #55, #56, #58, see Table 2 ▸) which are *locally isomorphic* to a five-dimensional cubic lattice within a ball of radius 10, as proven by isomorphism computations. Subgraph enumeration for #49, #52, #54, #55, #56, #58 has identified sets of three- as well as four-dimensional cubic lattices (last column of Table 2 ▸). These sets contain a finite number of connected components which interpenetrate each other in a manner as shown in Fig. 1 ▸. As a result, the above graphs could be built by interconnecting ‘layers’ of interpenetrating cubic lattices in a fourth dimension. Alternatively, they could be viewed as interconnected interpenetrating four-dimensional cubic lattices. Obviously both constructions imply Hopf links between quadrangular cycles. Qualitatively speaking, Hopf links arise from keeping the same amount (40) of quadrangular cycles per vertex while reducing the number of coordinate two-dimensional planes from 10 (in five dimensions) to 6 (in four dimensions). It is clear that quadrangular cycles of a five-dimensional cubic lattice lie separately in orthogonal two-dimensional coordinate planes and therefore are not linked. As a consequence, although being *locally isomorphic* to a five-dimensional cubic lattice, the above graphs are not locally isotopic to it. These examples illustrate perhaps a general phenomenon that knotting in crystal structures can formally arise from projections of high-dimensional periodic nets and represents a compromise of how a high-dimensional object could fit into a lower-dimensional space.

## Supplementary Material

Coordination sequences and point symbols. DOI: 10.1107/S2053273320007159/eo5107sup1.txt

Generating sets of enumerated Cayley graphs. DOI: 10.1107/S2053273320007159/eo5107sup2.txt

## Figures and Tables

**Table 1:** Vertex stabilizers and automorphism groups for enumerated Cayley graphs of 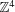 with valency 10 TD_15_ is the number of vertices in a ball of radius 15. D_*n*_ denotes a dihedral group of order *n*.

Graph	Stabilizer	TD_15_	BBNWZ†	Graph	Stabilizer	BBNWZ†	TD_15_
#1	C_2_ × S_3_ × D_8_	58321	20/22/1/1	#30	C_2_ × C_2_	2/3/2/1	165953
#2	C_2_ × C_2_ × S_4_	58321	25/11/2/1	#31	C_2_	1/2/1/1	185771
#3	C_2_ × C_2_ × S_3_	83521	14/10/1/1	#32	C_2_	1/2/1/1	220707
#4	C_2_ × C_2_ × S_3_	102631	14/10/4/1	#33	C_2_	1/2/1/1	205055
#5	C_2_ × C_2_ × C_2_	113915	4/4/3/1	#34	C_2_ × C_2_ × C_2_	4/4/6/1	158993
#6	C_2_ × S_4_	83521	24/5/4/1	#35	C_2_ × C_2_	2/3/2/1	180113
#7	C_2_ × S_4_	109001	24/5/1/1	#36	C_2_ × C_2_	2/3/2/1	198263
#8	C_2_ × S_4_	128111	24/5/3/1	#37	C_2_ × C_2_ × C_2_	4/4/5/1	173183
#9	D_12_	113915	8/5/1/1	#38	C_2_ × C_2_	2/3/2/1	192083
#10	D_12_	135937	8/5/1/1	#39	C_2_ × C_2_	2/3/2/1	207995
#11	D_12_	152239	8/5/1/1	#40	C_2_ × C_2_	2/3/2/1	212055
#12	D_12_	141943	8/5/1/1	#41	C_2_ × C_2_	2/3/2/1	176105
#13	D_12_	160533	8/5/1/1	#42	C_2_ × C_2_	2/3/2/1	189943
#14	D_12_	174063	8/5/3/1	#43	C_2_ × C_2_	2/3/2/1	195269
#15	C_2_ × C_2_ × C_2_	158773	4/4/5/1	#44	C_2_ × C_2_	2/3/2/1	198423
#16	C_2_ × C_2_	163723	2/3/2/1	#45	C_2_ × C_2_	2/3/2/1	214729
#17	C_2_ × C_2_	179453	2/3/2/1	#46	C_2_	1/2/1/1	202787
#18	C_2_ × C_2_	190703	2/3/2/1	#47	C_2_	1/2/1/1	216717
#19	C_2_ × C_2_	197363	2/3/2/1	#48	C_2_	1/2/1/1	215653
#20	C_2_ × S_5_	72601	31/7/1/1	#49	C_2_	1/2/1/1	235001
#21	C_2_ × C_2_ × S_3_	100811	14/10/2/1	#50	C_2_	1/2/1/1	209759
#22	C_2_ × C_2_ × S_3_	129931	14/10/6/1	#51	C_2_	1/2/1/1	218183
#23	C_2_ × C_2_ × C_2_	126213	4/4/5/1	#52	C_2_	1/2/1/1	235955
#24	C_2_ × C_2_	151733	2/3/2/1	#53	C_2_	1/2/1/1	212503
#25	C_2_ × C_2_	174503	2/3/2/1	#54	C_2_	1/2/1/1	229905
#26	C_2_ × C_2_ × C_2_	135475	4/4/6/1	#55	C_2_	1/2/1/1	227335
#27	C_2_ × C_2_ × C_2_	144913	4/4/5/1	#56	C_2_	1/2/1/1	234225
#28	C_2_ × C_2_	170843	2/3/2/1	#57	C_2_	1/2/1/1	223743
#29	C_2_ × C_2_	195155	2/3/2/1	#58	C_2_	1/2/1/1	238785

† The notation of four-dimensional space groups following Brown *et al.* (1978 ▸).

**Table 2:** Coordination sequences (up to the 15th sphere) for some Cayley graphs of 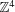 and a five-dimensional cubic lattice Coincident subsequences are highlighted in bold.

Graph	Coordination sequence	No. interpenetrating cubic lattices, 3D; 4D†
5D	10 50 170 450 1002 1970 3530 5890 9290 14002 20330 28610 39210 52530 69002	–
#49	**10 50 170 450 1002 1970 3530 5890 9290 14002 20330 28610** 37908 49238 62550	(2, 3); (3, 4, 5, 6, 7)
#52	**10 50 170 450 1002 1970 3530 5890 9290 14002 20330** 28318 38002 49616 63324	(3); (3, 4, 7, 9)
#54	**10 50 170 450 1002 1970 3530 5890 9290 14002 20330** 27798 36942 47838 60632	(2, 3); (2, 3, 5, 6, 7)
#55	**10 50 170 450 1002 1970 3530 5890 9290 14002** 20054 27472 36470 47192 59782	(3); (3, 5, 6, 7)
#56	**10 50 170 450 1002 1970 3530 5890 9290 14002 20330** 28144 37684 49112 62590	(2, 3); (2, 5, 6, 9)
#58	**10 50 170 450 1002 1970 3530 5890 9290 14002 20330 28610** 38530 50440 64510	(3); (3, 5, 7, 9)

† The notation (*a*, *b*, *c*, …) means that different subsets are possible which contain *a* (or *b*, or *c*, …) connected components.

## Footnotes

1 In actual computations, if a graph in question is a quotient of some other graph (as is the case for 10-valent Cayley graphs of  which are quotients of a five-dimensional hypercubic lattice, *cf*. Section 3), a good initial guess for *k* is the radius starting from which coordination sequences of a graph and its quotient become different. Then the described procedure for finding ρ(*r*) converges rather rapidly. For the graphs from Table 1 ▸ computations yield ρ(1) ≤ 13.

2 This program can also be accessed from the GAP package *Polyhedral* (Dutour Sikirić, 2015 ▸).

3 In this section, by a lattice we actually mean a Cayley graph of  for some standard generating set [*e.g*. for a (hyper)cubic lattice the set consists of *n* orthogonal unit vectors and their inverses].
